# Perceived Injury Risk among Junior Cricketers: A Cross Sectional Survey

**DOI:** 10.3390/ijerph14080946

**Published:** 2017-08-22

**Authors:** Prasanna J. Gamage, Lauren V. Fortington, Caroline F. Finch

**Affiliations:** 1 Australian Centre for Research into Injury in Sport and Its Prevention (ACRISP), Federation University Australia, SMB Campus, P.O. Box 663, Ballarat VIC 3353, Australia; l.fortington@federation.edu.au (L.V.F.); c.finch@federation.edu.au (C.F.F.); 2 School of Health Science and Psychology, Faculty of Health, Federation University Australia, SMB Campus, P.O. Box 663, Ballarat VIC 3353, Australia

**Keywords:** junior cricket, injury risk, risk perceptions, behaviours

## Abstract

Understanding how junior athletes perceive injury risks when participating in sport and the environment they play in is an important component of injury prevention. This study investigates how Sri Lankan junior cricketers (*n* = 365, aged 11–14 years, boys) perceive injury risks associated with playing cricket. The study used a Sri Lankan modification of an Australian junior cricket injury risk perception survey that considered playing cricket versus other sports, different cricket playing positions and roles, and different ground conditions. The risk of playing cricket was considered to be greater than that for cycling, but lower than that for rugby and soccer. Fast-bowlers, batters facing fast-bowlers, fielding close in the field, and wicket-keeping without a helmet were perceived to pose greater risks of injury than other scenarios. Playing on hard, bumpy and/or wet ground conditions were perceived to have a high risk opposed to playing on a grass field. Fielding in the outfield and wicket-keeping to fast-bowlers whilst wearing a helmet were perceived as low risk actions. The risk perceptions of junior cricketers identified in this study, do not necessarily reflect the true injury risk in some instances. This information will inform the development of injury prevention education interventions to address these risk perceptions in junior cricketers.

## 1. Introduction

Sport and recreational injuries among children are a significant health concern to society worldwide [1,2]. Adverse effects on physical health and well-being, long-term disability, financial burden to the family, and the effects on enjoyment in sports participation by juniors are some of the negative outcomes associated with sport injury [3,4,5]. Injuries during sports participation are associated with factors linked with the individual (e.g., age, gender, health, skills, physical fitness, previous injury and so forth), as well as external factors such as the environment, rules and adherence to rules, and sports governance [6]. Therefore, a broad, public health approach to sport injury prevention that recognises the wide range of contributing factors is essential for development of effective prevention measures. 

One area that has had little attention in sport injury prevention research is the contribution of injury risk perceptions. Risk perception refers to an individual’s personal judgment and evaluation of a risk or a danger that they might be exposed to [7]. The association between risk perceptions and decision making and subsequent actions are explained through psychological models such as the Health Belief Model [8]. According to this theory, individuals make a risk-benefit assessment based on the potential positive and negative outcomes of the proposed activity. In other words, the way a person perceives the risks will influence his/her behaviours and actions, where the higher the perceived risk, the greater the likelihood of avoiding the activity, and vice versa [9]. In this context, understanding risk perceptions will enable prioritising undesirable behaviours and adapt them into injury protective behaviours [9]. Making the decision to undertake risk-taking behaviour is multifactorial and includes both features associated with the activity or the danger itself, and also the unique characteristics of the individual, such as their attitude, beliefs and past experiences [7,10]. The model presented by Morrongiello and Lasenby-Lessard [11] describes individual characteristics, parent-family characteristics, and social-situational factors that determine risk-taking or avoidance behaviours. 

In the sports injury context, risk perception refers to the ability of an athlete to make an appropriate judgement of their own risk of sustaining an injury, and to modify their behaviours accordingly [12]. Risk-taking behaviours can be explained by heuristic psychological theories. Heuristics describe the methods that we use to make decisions and judgements quickly and efficiently under conditions of uncertainty or doubt [13,14]. As an example, a junior cricketer who believes the chance of sustaining an injury through a cricket ball hitting their head while batting is low, is more likely to play without a helmet. Only a few studies have looked at injury risk perceptions in sport, focusing on cricket [15,16], Australian football [17] and soccer [12]. One study in junior soccer athletes showed that injury risk perception was an important psychological risk factor for injury in adolescent sports, where a low perception of risk was equated to a significant increase in risk of injury [12]. A study conducted among junior Australian footballers showed, despite a clear understanding of later life consequences and importance of proper rehabilitation of injuries, most players believed that they should take the risk and play while they are injured [17]. Thus, injury risk perceptions of junior athletes can be a useful precursor to understanding subsequent behaviours and decisions, thereby informing the design of sport safety measures from early stages of sports participation. 

The Juniors Enjoy Cricket Safely (JECS) study was an Australian project established to identify injury patterns, injury risk perceptions and playing behaviours in order to promote injury prevention and safe participation in cricket [15,16,18,19]. White and colleagues [15] reported the injury risk perceptions of Australian junior cricketers, and this remains the only study of injury risk perceptions among junior cricketers in the international peer-reviewed literature. That study showed that Australian junior cricketers perceived highest risk when fielding in close to the batter, and batting facing a fast bowler [15]. Overall recommendations were mainly focused on highlighting the role of the coach in implementing preventive strategies in the identified areas of undesirable risk perceptions. These include continual reminding of the importance of safety strategies such as protective gear, improving techniques and skills, and providing accurate information on injury risks to the junior cricketers [15]. 

Cricket is the most popular sport in Sri Lanka, especially among children and adolescents, where there are large numbers of participants at both competitive and recreational levels [20]. Compared to other developed western cricket playing nations, Sri Lankan junior cricketers represent different socio-economic and cultural traits, with potential disadvantages in their knowledge and resource availability for injury prevention and risk management [21]. In addition, the standards of the playing environment for cricket (e.g., ground conditions) and climatic risks associated with hot and humid conditions in Sri Lanka can also create an added risk. These differences in environmental, social, cultural and economic qualities can have a potential impact on risk perceptions and risk behaviours among junior athletes [11,22]. Therefore, the primary objective of this study is to investigate how Sri Lankan junior, male cricketers perceive their risk of injury when playing cricket. The study provides an opportunity to compare risk perceptions in the Sri Lankan cohort with those from Australia, two socio-economically and culturally different cricket populations.

## 2. Materials and Methods 

### 2.1. Participants

This study was designed as a descriptive cross-sectional survey. All junior school cricketers in the boys under-13 years age category, who took part in the 2016 inter-district cricket tournament across Sri Lanka were invited to participate in the study. This included 48 teams in total, representing all nine provinces in Sri Lanka. The survey was carried out in collaboration with the Sri Lanka School Cricket Association (SLSCA), and data collection was facilitated by the district cricket team coaches. Ethical approval was obtained from the Ethical Review Committee of (A16-039). Information detailing the aim of the study, data collection procedure, benefits and potential risks were provided to, and subsequently informed written consent was obtained from, both players and their parents.

### 2.2. Development of the Survey Questionnaire

The Australian JECS injury risk perception questionnaire was used as the survey self-report questionnaire instrument [16]. This questionnaire was developed, validated and tested among Australian junior cricketers to identify their injury risk perceptions associated with playing cricket [15]. Face and content validity of this questionnaire was evaluated through expert feedback [16]. Components of the original English version of the questionnaire that were not related to injury risk perceptions were removed (e.g., other sports participation). The questionnaire was then modified to suit the Sri Lankan context and setting by the lead author (e.g., the question “I want to play cricket for Australia” was modified to “I want to play cricket for Sri Lanka”; a response option of “Australian football” was replaced by a local sport “elle”).

The adapted English version of the questionnaire was then translated into Sinhala and Tamil languages, which are the two main languages in Sri Lanka. The translated Sinhala and Tamil versions of the questionnaires were then examined by two Sinhala and Tamil language school teachers, for language accuracy (grammar and spelling mistakes), clarity and age-appropriateness. Both the Sinhala and Tamil questionnaires had 88–100% agreement for questions on demographic data and participation in cricket, and injury history. Out of all injury risk perception questions, 73% of Sinhala and 90% of Tamil questions showed a substantial (Kappa value = 0.61–0.8) and almost perfect (Kappa value = 0.81–1.0) agreement, suggesting a strong reliability. The full translation process and reliability testing will be reported in a separate paper. The final survey, JECS-Sri Lanka (JECS-SL), was administered to the junior cricketers. 

The JECS-SL questionnaire consisted of three parts. Part A focused on demographics (e.g., age, gender) and participation in cricket (e.g., playing experience). Part B asked about the players” worst injury during the last cricket season. The first section of Part C inquired about how junior cricketers perceived the risk of injury in different scenarios, when (1) playing cricket versus other sports, (2) engaging in different cricket tasks by player position (bowling, batting, fielding and wicket-keeping), and (3) playing in different ground conditions. The second section of Part C inquired about the rules and regulations for safe participation in cricket. The different sports included in injury risk perception assessment in this study were classified as collision (rugby), contact (soccer, basketball), and limited contact (cycling, elle and cricket) for data analysis and presentation [23]. Risk perceptions were measured on a 3-point Likert scale (“no chance”, “a small chance”, “a high chance”), with an additional response option of “don’t know”. 

### 2.3. Data Collection Method

The data collection was carried out during the 2016 school cricket season running from October to December. At the beginning of the tournament, fifteen sets of questionnaires in all three languages (Tamil, Sinhala and English) were posted to each team coach of the selected 48 district teams. Fifteen questionnaires were given for the first eleven members of the team, with an extra four questionnaires for any reserve players. Players chose a questionnaire in their preferred language, and written instructions were given detailing how to fill them in. Players were asked to complete the questionnaires anonymously after obtaining consent from their parents. Completed questionnaires were collected by the team coaches and returned to the research team via post. To maximize the response rate, the district team coaches were given reminders at 4 weeks into the data collection through an email, and at 8 weeks through a phone call. Data collection was completed at the end of the tournament in December. In order to protect participants” confidentiality, each questionnaire was distributed in a separate envelope that was to be sealed on return.

### 2.4. Statistical Analysis 

**Figure 1:**
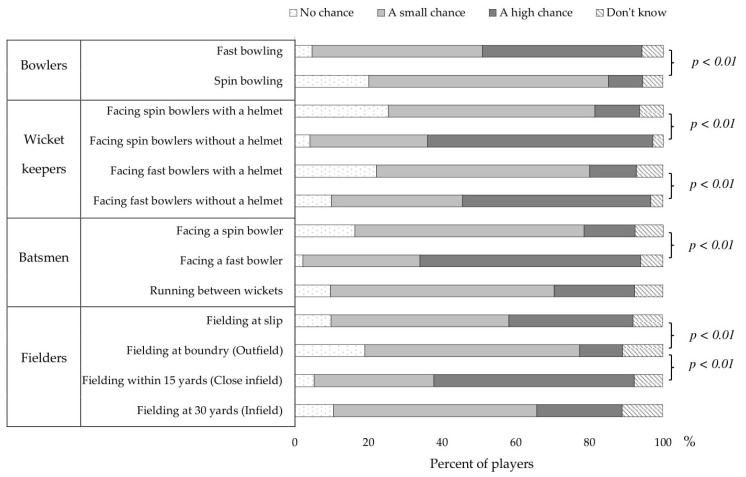
Injury risk perceptions in cricket-specific tasks at different playing positions. *Note*: *p* values were calculated comparing the median scores between selected response variables using Wilcoxon signed rank test.

Collected surveys were coded and entered into an excel database for initial editing, then exported to SPSS Version 24 (IBM Corp., Armonk, NY, USA) for statistical analysis. Demographic data were analysed using descriptive statistics (mean and standard deviation). Responses to the Likert-scale risk perception questions were analysed as frequencies (%) with 95% confidence interval (CI). The three valid response categories in the Likert scale questions were coded as “no chance = 1”, “a small chance = 2”, “a high chance = 3”, and the collective mean was calculated for each response. The median scores were compared across questions of interest (between *Cricket-Self* and *Cricket-Other* (Table 1), between *Cricket* and *Other sports* (Table 2), and between different *Cricket specific tasks* (Figure 1)) using Wilcoxon signed rank tests with a significance level of *p* < 0.05. “Don’t know” responses were removed before calculating the median scores.

## 3. Results

A total of 720 questionnaires were distributed among the 48 district junior cricket teams from which 33 teams (out of 48, team response rate = 69%) returned the questionnaires. There was at least one team from each of the nine provinces in Sri Lanka, resulting in country-wide representation. Out of 720 possible participant responses, 365 questionnaires were returned (Sinhala = 251; Tamil = 114; English = 0), achieving an overall player response rate of 51%, and 11 participants returned surveys per team, on average (range = 7 to 15). The mean age of the players was 12.9 ± 0.9 years and they had been playing competitive cricket (school cricket) for 2.6 ± 1.8 years on average. 

Injury risk perceptions while participating in cricket and sports in general are summarised in Table 1. Nearly half of the survey respondents (164; 45.3%) believed that there is a chance of themselves getting injured while participating in any sport. When playing cricket specifically, injury risk to themselves *(Cricket-Self)* were perceived as follows: 4.9% no chance, 49.7% a small chance, 26.9% a high chance, and 18.4% don’t know. The perceived mean risk of injuries to themselves *(Cricket-Self)* when playing cricket was almost similar to the perceived mean risk of injury to other players *(Cricket-Other)* in general (*p* = 0.234). 

Responses of perceived injury risk for cricket and five other sports are presented in Table 2. Cricket (29.9%), basketball (25.9%), cycling (26.6%) and elle (18.8%) had lower percentages for having “a high chance” of getting injured than soccer (58.8%) and rugby (72.6%). Compared to cricket (limited contact sport), participants in this study perceived rugby (collision sport; *p* < 0.001) and soccer (contact sport; *p* < 0.001) as having a greater risk for injuries. However, the risk of injury when playing cricket was perceived to be higher than that in other limited contact sports (cycling and elle; *p* < 0.001). In the limited-contact sports group, the players perceived cycling (14.6%) and elle (21.8%) to have “no chance” of getting injured, compared to 5.2% in cricket.

The perceived injury risk percentage scores when engaging in specific cricket tasks for different playing positions (bowling, batting, fielding and wicket-keeping) are presented in Figure 1. Fast-bowlers were identified as a group with a “high chance” of getting injured (43.3%) compared to spin-bowlers (9.3%; *p* < 0.01). Similarly, batters facing fast-bowlers were considered to have a “high chance” of getting injured (59.9%) compared to batters facing spin-bowlers (13.8%; *p* < 0.01). Most junior cricketers believe the chance of a batter getting injured when running between wickets is “small” (60.8%). 

Survey respondents perceived fielding in the outfield as safer than fielding in the infield (i.e., when closer to the batter). Injury risk perceptions associated with wicket-keeping-related tasks were related to both the type of bowler faced with and helmet use. The chance of wicket-keepers getting injured while not wearing a helmet was perceived as “high” when facing a spin-bowler (61.2%) and a fast-bowler (51.1%). When wearing a helmet, wicket-keepers were mainly perceived as having “no chance” (25.4% spin-bowler vs. 22.2% fast-bowler) or a “small chance” (56.1% spin-bowler vs. 57.9% fast-bowler) of injury.

Injury risk perception when playing cricket on different ground conditions is presented in Table 3. Most survey respondents reported “no chance” (21.9%) or a “small chance” (62.7%) of getting injured when playing on grass. This is in contrast to perceptions in relation to other ground conditions, where most junior cricketers believe that there is a “high chance” of getting injured when playing on a hard ground (74.2%; mean = 2.75, 95% CI 2.70, 2.80), on a bumpy ground (76.6%; mean = 2.80, 95% CI 2.76, 2.85), on a wet ground (54.7%; mean = 2.54, 95% CI 2.48, 2.60), or playing when it’s raining (62.9%; mean = 2.68, 95% CI 2.63, 2.74).

With reference to the rules and regulations for safe participation in cricket, 329 (91.6%) of the junior cricketers suggested that rules to do with wearing safety gear (helmets, pads) were the most important. More than one third (39%) believed that there should be “more rules to make playing junior cricket safer”. In contrast, 38.5% believed there is “no need for any more rules” to do with safety, and 22.5% believe that there are “too many rules in junior cricket” to do with safety.

## 4. Discussion

This study evaluated injury risk perceptions among Sri Lankan junior cricketers using a translated and culturally adapted version of the JECS injury risk perception questionnaire, originally developed for the Australian junior cricket context. This is the first study to examine injury risks among Sri Lankan junior cricketers, and also to use the JECS questionnaire outside of Australia. Overall, the findings of both Australian and Sri Lankan studies showed common injury risk perception patterns [15]. For example, both groups believed fielding closer to the batter (as opposed to fielding in the outfield) and batting facing a fast-bowler (as opposed to facing a spin-bowler) have a greater risk of getting injured. In terms of preventing injuries and providing a safer playing environment for junior cricketers, the present study further emphasises the need of providing information to junior cricketers about safety strategies and careful monitoring of the safety measures. 

The Australian JECS injury risk perception study by White et al. [15] was designed as a self-reported cross-sectional survey, similar to the present study. They recruited 284 participants registered with junior clubs from the Ballarat-Victoria under three age categories (under 12, 14 and 16; age = 8–16 years). The questions in the translated JECS-SL questionnaire was very similar to the original JECS questionnaire, with exception of minor modifications addressing cultural/language differences and the addition of five questions illustrating more playing scenarios. The directly comparable questions provided similar responses as those obtained from the Australian study by White et al. [15]. This similarity was most apparent when comparing the under 13 Sri Lankan cohort with the under 12 and under 14 cohorts from Australia (i.e., the same age category as the present study). 

The present study shows that the perceived injury risk by junior cricketers of getting injured themselves was similar to another person getting injured when playing cricket. This observation among Australian junior cricketers [15], and other junior sports [24], showed that players underestimate their own risk perhaps due to optimism bias [25]. Optimism bias explains the tendency of an individual to underestimate the possibility they will experience an adverse event (i.e., assume more favourable outcomes for the self) [26]. Optimism bias positively correlates with subjective invulnerability (i.e., an individual tends to think that he/she is incapable of being injured or harmed), convincing a person to engage in a high-risk behaviour or task [27]. This likelihood of risk taking has the potential to be modified, and its consequences can be minimised through educating players on the importance of safety strategies by parents, coaches and other support staff. 

The different sports included in the survey are generally played in most schools in Sri Lanka, and are popular among junior athletes. As expected for comparison purposes, the injury risk when playing rugby and soccer was perceived as being higher than that when playing cricket. This is likely due to direct body contact nature in rugby and soccer, compared to minimal or non-body contact nature in cricket. This observation is supported by evidence, where the highest injury rates have been reported in collision-type sports, such as ice hockey, rugby and soccer [28,29]. On the other hand, junior cricketers believed there to be a higher risk of getting injured in playing cricket compared to limited contact (cycling, elle) sports. Rating different sports based on the injury risk may not be an accurate reflection of absolute injury risk. For example, one junior cricketer may believe cricket is a safer sport than other sports, as cricketers wear protective gear when playing; another junior cricketer may believe cricket is a dangerous sport if he/she had suffered a serious injury in the past when playing cricket. Therefore, it could be useful to recognise the exact reasons for specific risk perceptions of different sports and assist them in taking appropriate safety decisions. 

With respect to injury risk perceptions in different cricket specific tasks, fast-bowlers were perceived to be at a significantly greater risk of injury than spin-bowlers. A greater perceived risk in fast-bowling can be explained by two probable concepts. Firstly, compared to spin-bowlers, fast-bowlers require a longer and faster run up, forceful and dynamic bowling action, and stronger physique. As explained by the representativeness heuristic model, this explosive nature of fast-bowling would enable junior cricketers to visualize the greater risk associated with fast-bowling [13]. Secondly, the news and discussions telecast on media, often highlights the major injuries to role model fast-bowlers and them missing crucial matches due to injury, which might also influence junior risk perceptions.

At the elite level, injury incidence among batters and fielders is 2–3 times lower than that in fast-bowlers [30,31,32]. In light of current evidence from junior cricket, injury prevalence among batters and fielders have been reported to be similar or higher than in bowlers [18]. In the areas of batting and fielding, both the Sri Lankan and Australian junior cricketers [15] identified two specific cricket tasks as having greatest injury risk: (1) batters facing fast-bowlers, and (2) fielding closer to the batter. Batters struck by the cricket ball while facing a fast-bowler is the main injury mechanism in junior cricket batting [18,33], suggesting Sri Lankan junior cricketers have identified risk perceptions accurately. The questions related to fielding tasks in the present study were expanded from the original JECS questionnaire, in order to provide a more specific description of fielding positions (fielding at the boundary line, at the 30-yard circle, within 15-yard, at slips). Despite these modifications, both studies reported similar findings in that a greater injury risk was perceived for fielding closer to the batter and lesser risk fielding in the outfield. The current evidence on fielding injuries among junior cricketers suggest that injury risk is commonly related to misfielding or mishandling the ball [18]. While no association with fielding position or proximity to the batter has been identified, this has not been investigated specifically. More research to provide the supporting evidence on injury risk is needed in this population.

Facial injuries among wicket-keepers are a common concern because catastrophic eye and dental injuries have been previously reported at the international level [34,35]. Injury data suggest that wicket-keepers are at a greater risk of injuries facing fast-bowlers than spin-bowlers [36]. In the present study, injury risk was perceived as “greatest” when keeping wickets without wearing a helmet, regardless of bowling type. This highlights the fact that junior cricketers acknowledge the importance of wearing helmets to prevent injuries. Interestingly, perceived injury risk was greater for facing a spin-bowler than for facing a fast-bowler, when not wearing a helmet. In present-day cricket, wicket-keepers often wear helmets for both fast-bowlers and spin-bowlers when they stand closer to the wicket, but usually do not wear helmets when they stand back to the fast-bowlers. Therefore, this incongruity of judgement is most likely due to not specifying the standing position of the wicket-keeper in the questions.

The effects of ground hardness in sustaining injuries in sports is inconclusive [19]. In relation to ground hardness, the findings of the present study are in agreement with what has been observed among Australian junior cricketers, where a majority perceived a “high chance” of getting injured when playing on a hard and bumpy ground compared to playing on grass [15]. Cricket is a summer sport in Australia, and is mainly played during the hot-dry season. In contrast, Sri Lanka has a tropical climate with wet and dry seasons, and cricket is being played throughout the year. Therefore, playing in the rainy season on wet ground conditions is a common experience for cricketers in Sri Lanka. Two new questions related to this area (“playing when it’s raining” and “playing on a wet ground”) were included in the JECS-SL questionnaire that were not assessed in the Australian study [15]. More than 50% of cricketers believed that there is a “high chance” of getting injured when playing in these conditions, but a considerable proportion (33.7% and 23.6%) thought the chance was “small”. At present, there is no information on the quality and standards of ground conditions and playing surfaces in Sri Lankan set up. In Sri Lankan school cricket, the match umpire assesses the ground conditions and hazards, and decision on the safety and suitability of playing surface is made on his/her subjective evaluation [37]. Information obtained from this study on the risk perceptions related to ground conditions are likely to benefit and can be used to highlight the areas that need attention in developing effective injury prevention policies and guidelines for the Sri Lankan cricket context in future.

Interventions that can be used to modify and change undesirable risk perceptions into more accurate risk perceptions can be expected to subsequently change safety behaviours [9]. Therefore, the knowledge and understanding of the risk perceptions of Sri Lankan junior cricketers gained from the present study will be able to be translated into successful injury prevention interventions. In a setting where safety behaviours of the players have already been recognized or known, more specific and targeted preventive interventions can be developed [9]. Such safety behaviour interventions have been shown to reduce the injury rates significantly among Australian junior cricketers in relation to helmet use during cricket batting [33]. For Sri Lanka, this study is the first to examine injury risk perceptions of any nature among junior cricketers. Identifying the actual safety behaviours of Sri Lankan junior cricketers in future will enable the linking of knowledge gathered here to deliver effective preventive interventions, and also provide directions for future research in this area. Similar to the directions provided by White et al. [15] for Australian junior cricketers, our study informs different strategies that can assist in developing injury prevention interventions specific to the Sri Lankan context. Future culturally relevant actions to educate the players, parents and coaches to modify negative risk perceptions and attitudes, while supporting positive risk perceptions, will benefit all those involved in cricket in Sri Lanka. 

### Strengths and Limitations

We obtained survey responses from all nine provinces of Sri Lanka, achieving a representative sample with country-wide participation. An overall team response rate of 69% and individual response rate of 51% (based on the maximum number of questionnaires that were sent out) was obtained with an average of 11 participant responses per team. This response rate is considered as very strong for a study of this nature.

The original JECS-Australia questionnaire was developed and tested among a wider age group of junior cricketers (10–16 years). The present study was limited to junior cricketers in the under-13 age category only. For feasibility in collecting data, we included the players who took part in the inter-district tournament, and data collection was facilitated by their team coaches. The district teams represent only the players who have performed well as cricketers in that particular district and therefore all other school cricketers in those districts were not included in this study. Our survey used a 3-point Likert scale because that is what the original JECS survey used. This may have reduced the ability to detect variation across the sample.

## 5. Conclusions

Sri Lankan junior cricketers were mostly accurate and logical in their injury risk perception ratings, particularly in the areas of bowling (fast-bowling vs. spin-bowling), cricket batting (against fast-bowlers vs. spin-bowlers) and wicket-keeping in relation to helmet use. They perceived the injury risk as “low”, when fielding in the infield (at 30-yard) and outfield (at the boundary), opposed to fielding in the close-infield closer to the batter (within-15 yard), which results are consistent with previous data from Australian junior cricketers. Recognised injury risk perceptions of junior cricketers in this study may not reflect the actual injury risk in some instances. This highlights the necessity of educating and supporting junior cricketers, in modifying their perceived risk attitudes and beliefs, based on research evidence in cricket. This study provides new data from a socio-economically and culturally different junior cricket population to that previously studied but were very similar to risk perception behaviours and attitudes from Australian junior cricketers. 

### Practical Implications

- Sri Lankan junior, male cricketers perceived a greater risk of injuries when fast-bowling, batting facing fast-bowlers, fielding close in the infield, and wicket-keeping without a helmet than other scenarios.
- Injury risk perceptions among Sri Lankan junior cricketers are similar to those reported among Australian junior cricketers for most of the surveyed playing situations and conditions.
- Injury risk perceptions might not reflect the actual injury risk in some instances, however, more research to provide the supporting evidence on injury risk is needed in this population.

## Figures and Tables

**Table 1:** Frequency and percentage (%) of perceived injury risk for self and others when playing sport, and when playing cricket.

**Chance of Getting Injured (*n* = 362)**									
Question: Chance of getting injured	Yes	No	Not sure		Missing responses				
When playing some sports?	164 (45.3%)	73 (20.2%)	125 (34.5%)		3				
**Chance of Getting Injured (*n* = 364)**									
	**No Chance**	**Small Chance**	**High Chance**	**Don’t Know**	**Missing Responses**	**Mean ± SD ^†^**	**95% CI ^†^**	**Median (Range)**	***p*** Value *
When playing cricket yourself? *(Cricket-Self)*	18 (4.9%)	181 (49.7%)	98 (26.9%)	67 (18.4%)	1	2.27 ± 0.6	2.20, 2.33	2 (1–3)	0.234
For a person playing cricket? *(Cricket-Other)*	19 (5.2%)	206 (56.6%)	109 (29.9%)	30 (8.2%)	1	2.27 ± 0.6	2.21, 2.33	2 (1–3)	

^†^ Mean ± SD calculated for each response variable with 95% CI, after removing the “don’t know” responses; * Results derived from comparing median scores between *Cricket-Self* and *Cricket-Other* using Wilcoxon signed rank test.

**Table 2:** Frequency and percentage (%) of perceived risk, a person has of getting injured among different sports, and comparison of scores with playing cricket using Wilcoxon signed rank test.

			Chance of Getting Injured						
Sport	No Chance	Small Chance	High Chance	Don’t know	Missing Responses	Mean ± SD ^†^	95% CI ^†^	Median (Range)	*p* Value *
Rugby (*n* = 365)	3 (0.8%)	65 (17.8%)	265 (72.6%)	32 (8.8%)	0	2.79 ± 0.4	2.74, 2.83	3 (1–3)	<0.001
Soccer (*n* = 362)	6 (1.7%)	111 (30.7%)	213 (58.8%)	32 (8.8%)	3	2.63 ± 0.5	2.57, 2.68	3 (1–3)	<0.001
Cricket (*n* = 364)	19 (5.2%)	206 (56.6%)	109 (29.9%)	30 (8.2%)	1	2.27 ± 0.6	2.21, 2.33	2 (1–3)	
Basketball (*n* = 363)	32 (8.8%)	177 (48.8%)	94 (25.9%)	60 (16.5%)	2	2.20 ± 0.6	2.14, 2.27	2 (1–3)	0.379
Cycling (*n* = 364)	53 (14.6%)	175 (48.1%)	97 (26.6%)	39 (10.7%)	1	2.14 ± 0.7	2.06, 2.21	2 (1–3)	0.001
Elle ^‡^ (*n* = 362)	79 (21.8%)	163 (45%)	68 (18.8%)	52 (14.4%)	3	1.96 ± 0.7	1.89, 2.04	2 (1–3)	<0.001

^†^ Mean ± SD calculated for each response variable with 95% CI, after removing the “don’t know” responses; * Results derived from comparing median scores between *Cricket* and *Other sports* using Wilcoxon signed rank test; ^‡^ A bat and ball type local sport in Sri Lanka.

**Table 3:** Frequency and percentage (%) of perceived injury risk when playing in different ground conditions, and mean of risk with 95% confidence interval (CI).

	Chance of Getting Injured						
Ground Condition	No Chance	Small Chance	High Chance	Don’t Know	Mean ± SD *	95% CI *	Median (Range)
Playing on a bumpy ground (*n* = 364)	6 (1.6%)	55 (15.1%)	279 (76.6%)	24 (6.6%)	2.80 ± 0.4	2.76, 2.85	3 (1–3)
Playing on a hard ground (*n* = 365)	7 (1.9%)	73 (20%)	271 (74.2%)	14 (3.8%)	2.75 ± 0.5	2.70, 2.80	3 (1–3)
Plying when it’s raining (*n* = 364)	8 (2.2%)	86 (23.6%)	229 (62.9%)	41 (11.3%)	2.68 ± 0.5	2.63, 2.74	3 (1–3)
Playing on a wet ground (*n* = 362)	16 (4.4%)	122 (33.7%)	198 (54.7%)	26 (7.2%)	2.54 ± 0.6	2.48, 2.60	3 (1–3)
Playing on grass (*n* = 365)	80 (21.9%)	229 (62.7%)	26 (7.1%)	30 (8.2%)	1.84 ± 0.5	1.78, 1.90	2 (1–3)

* Mean ± SD calculated for each response variable with 95% CI, after removing the “don’t know” responses.
